# Frequency and Significance of Abnormal Pancreatic Imaging in Patients with BRCA1 and BRCA2 Genetic Mutations

**DOI:** 10.1155/2016/5619358

**Published:** 2016-03-16

**Authors:** Elie Chahla, Antonio Cheesman, Suzanne M. Mahon, Robert W. Garrett, Ben P. Bradenham, Theresa L. Schwartz, Louay Omran, Jason R. Taylor, Samer Alkaade

**Affiliations:** ^1^Division of Gastroenterology and Hepatology, Saint Louis University School of Medicine, St. Louis, MO 63110, USA; ^2^Department of Internal Medicine, Saint Louis University School of Medicine, St. Louis, MO 63110, USA; ^3^Division of Hematology and Oncology, Saint Louis University School of Medicine, St. Louis, MO 63110, USA; ^4^Department of Radiology, Saint Louis University School of Medicine, St. Louis, MO 63110, USA; ^5^Department of General Surgery, Saint Louis University School of Medicine, St. Louis, MO 63110, USA

## Abstract

*Objective.* Pancreatic adenocarcinoma is typically diagnosed in advanced stages resulting in a significant reduction in the number of patients who are candidates for surgical resection. Although the majority of cases are believed to occur sporadically, about 10% show familial clustering and studies have identified an increased frequency of BRCA germline mutations. The role of screening for pancreatic adenocarcinoma in these populations is unclear. Our study aims to identify the abnormal pancreatic imaging findings in BRCA1 and BRCA2 mutation carriers.* Methods.* A retrospective review of patient medical records with known BRCA1 and BRCA2 mutations was conducted. Data was collected and all available abdominal imaging studies were reviewed.* Results.* A total of 66 patients were identified, 36 with BRCA1 and 30 with BRCA2 mutations. Only 20/66 (30%) had abdominal imaging (14 BRCA1 and 6 BRCA2 patients). Of those patients with abdominal imaging, abnormal pancreatic imaging findings were detected in 7/20 (35%) cases.* Conclusion.* Our study shows a high incidence of abnormal pancreatic imaging findings in patients with BRCA genetic mutations (35%). Larger studies are needed to further define the role of pancreatic cancer screening and the significance of abnormal imaging findings in BRCA1 and BRCA2 mutation carriers.

## 1. Introduction

Pancreatic ductal adenocarcinoma (PDAC) is the fourth leading cause of cancer death in the United States and contributes to an estimated 227,000 deaths per year worldwide [1]. It is estimated that PDAC will become the second leading cause of cancer-related death by the year 2030 [2]. Due to its concealed nature and lack of specific clinical findings in the early stages, typically only 10–20% of patients are surgical resection candidates at time of diagnosis and 5-year survival rates are as low as 6.9% [3, 4]. Despite its dismal prognosis, currently there is insufficient data to indicate that screening for PDAC is effective in reducing morbidity and mortality. As a result, guidelines recommend against general population screening [5]. Although recent consensus panels suggest potential benefits for PDAC screening in high-risk populations, further characterization of such individuals is needed [6].

Abdominal imaging studies, including transabdominal ultrasound (US), computed tomography (CT), magnetic resonance cholangiopancreatography (MRCP), and endoscopic ultrasound (EUS), are the most widely used techniques for identification of pancreatic neoplasia. However, their benefit in screening high-risk individuals is inconclusive.

Most cases of PDAC are believed to occur sporadically; however as many as 10% show familial clustering.

This is defined by the presence of PDAC in two first-degree relatives or a total of three or more non-first-degree relatives [7]. In addition, twin studies have shown heritable factors may account for up to 35% of cases [8]. Research studies comparing familial PDAC clusters reveal that the most common germline mutation is BRCA2 (6–17%) [9–11]. Nonfamilial cluster based reports also indicate an increased risk for PDAC in BRCA1 (RR 2.26) and BRCA2 (RR 3.51) mutation carriers [12–16].

Despite the evidence supporting BRCA2 and BRCA1 as risk factors for PDAC, there is no data on the frequency of abnormal pancreatic imaging in carriers of these genetic mutations. Our study aims to identify the frequency of abnormal pancreatic imaging findings in patients with BRCA1 and BRCA2 genetic mutations.

## 2. Methods

We conducted a retrospective medical record review of patients who tested positive for known at-risk BRCA1 and BRCA2 mutations by genetic sequencing. The study was performed at a tertiary level academic center and included patients who were tested between January 2006 and July 2014. Data regarding personal and first-degree relative familial history of cancer was obtained. All existing abdominal imaging CT studies were reviewed by the same gastrointestinal radiologist (RG). EUS was performed by two therapeutic endoscopists (SA, JT). The abdominal imaging modalities examined included CT and/or EUS. All abnormal pancreatic imaging findings were reported. Strict criteria were used to diagnose pancreatic atrophy both on cross-sectional imaging and endoscopically.

## 3. Results

**Figure 1:**
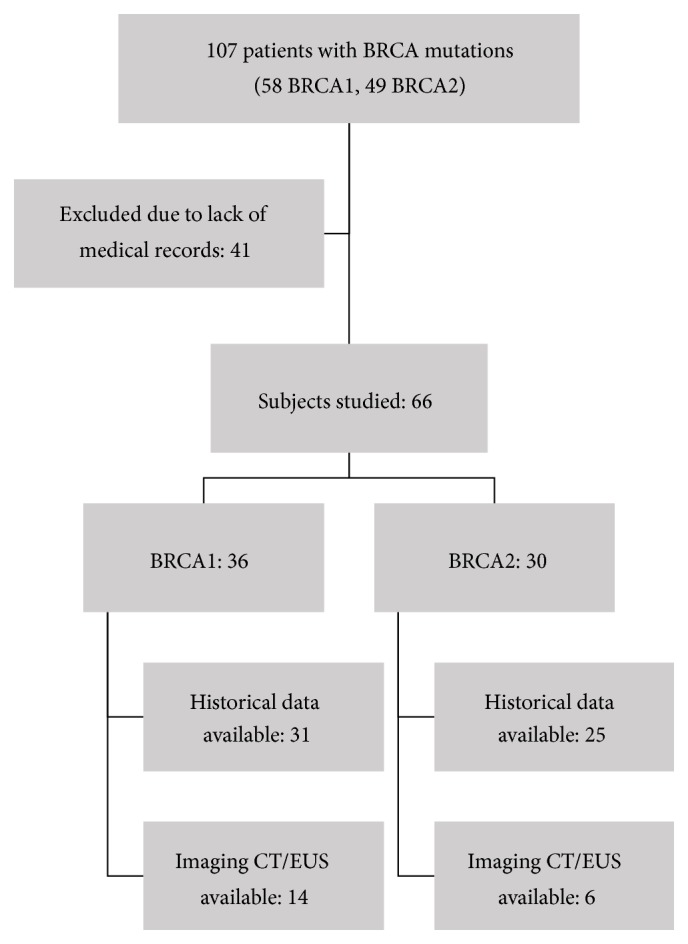
BRCA1/BRCA2 germline mutation carriers, historical data, and imaging studies available.

A total of 107 patients with BRCA deleterious mutations were identified (58 BRCA1, 49 BRCA2). Only 66 subjects from the original group had available medical records (36 BRCA1, 30 BRCA2); the remaining 41 patients had been referred for genetic testing and counseling without further workup. Detailed personal and family history of cancer was reported for 31 BRCA1 and 25 BRCA2 patients from those with available records. Abdominal imaging studies were available for only 20 patients (14 BRCA1, 6 BRCA2) (Figure 1).

**Figure 2:**
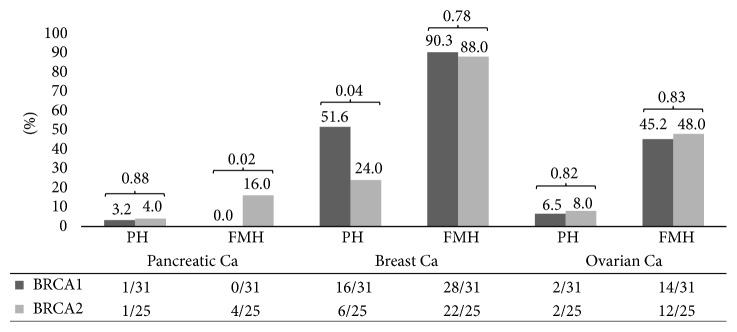
BRCA1/BRCA2 mutation carriers personal and familial history of cancer. Percentages noted in chart and absolute frequencies noted in table. PH: personal history, FMH: family history.

One subject in each BRCA class had a confirmed diagnosis of PDAC. Family history of PDAC was more prominent in patients carrying the BRCA2 mutation (16%). Other personal and familial history was notable for breast and ovarian cancer in both BRCA1 and BRCA2 patients (Table 1, Figure 2).

**Figure 3:**
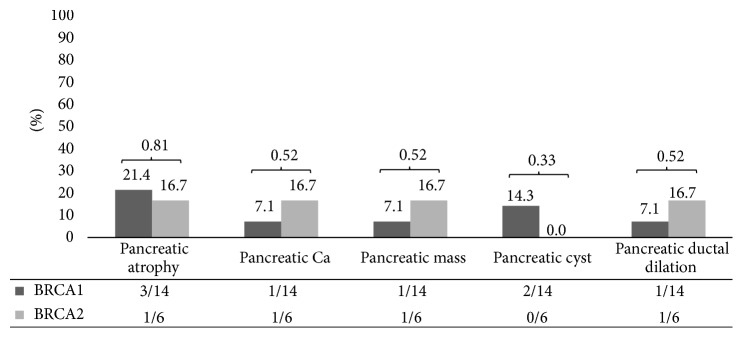
BRCA1/BRCA2 mutation carriers and pancreatic imaging findings. Percentages noted in chart and absolute frequencies noted in table.

Review of abdominal imaging studies available (*n* = 20) revealed abnormal pancreatic imaging findings in 7/20 (35%) cases. This includes pancreatic atrophy in 3 BRCA1 (21.4%) patients and 1 BRCA2 (16.7%) patient, mass lesions later identified as pancreatic cancer in 1 BRCA1 (7.1%) patient and 1 BRCA2 (16.7%) patient, pancreatic cysts in 2 BRCA1 (14.3%) patients, and pancreatic ductal dilation in 1 BRCA1 (7.1%) patient and 1 BRCA2 (16.7%) patient (Table 2, Figure 3).

Only patients with BRCA2 germline mutations had first-degree family members with PDAC. This subgroup of patients however failed to demonstrate any identifiable abnormal pancreatic imaging findings. In addition, both patients with known personal history of PDAC had no family history of the disease.

## 4. Discussion

The low absolute risk for development of PDAC in the general population, lack of cost-effective testing, and the increased risk for absolute harm from unnecessary procedures preclude general population screening [17]. Expert recommendations from the “International Cancer of the Pancreas Screening (CAPS) Consortium” advocate screening for PDAC in high-risk populations.

*High-Risk Individuals as Defined by the International Cancer of the Pancreas Screening (CAPS)*

1. Individuals with three or more affected blood relatives, with at least one affected FDR, should be considered for screening.
2. Individuals with at least two affected FDRs with PC, with at least one affected FDR, should be considered for screening once they reach a certain age.
3. Individuals with two or more affected blood relatives with PC, with at least one affected FDR, should be considered for screening.
4. All patients with Peutz-Jeghers syndrome should be screened, regardless of family history of PC.
5. p16 carriers with one affected FDR should be considered for screening.
6. BRCA2 mutation carriers with one affected FDR should be considered for screening.
7. BRCA2 mutation carriers with two affected family members (no FDR) with PC should be considered for screening.
8. PALB2 mutation carriers with one affected FDR should be considered for screening.
9. Mismatch repair gene mutation carriers (Lynch syndrome) with one affected FDR should be considered for screening.

The above list is adapted from [6].

The main risk factors include patients with strong family history of PDAC and certain genetic mutations with or without affected family members. The genetic mutations and syndromes that are linked to PDAC include Peutz-Jeghers syndrome, Lynch syndrome, p16 carriers, BRCA2 mutation, and PALB2 mutation carriers.

There was agreement on the screening modalities, which included EUS and/or MRI/MRCP. However, there was no consensus on when to initiate or end screening and on the interval duration between tests [6].

Multiple reports have previously shown an increased risk for PDAC in patients with BRCA germline mutations. However to date, no studies have commented on the frequency and significance of abnormal pancreatic imaging findings in these patients.

Abnormal pancreatic imaging findings including pancreatic atrophy, cysts, ductal dilation, or cancer were identified in 35% of patients with known at-risk BRCA1 and BRCA2 genetic mutations. No statistical significant differences were found in the occurrence of abnormal imaging findings between BRCA1 and BRCA2 groups.

Family history of pancreatic cancer was most prominent in patients with BRCA2 germline mutations. Although this supports previous expert recommendations from the “CAPS Consortium” to consider pancreatic cancer screening of BRCA2 mutation carriers with family history of the disease [6], it questions the methods and efficacy for such screening. The lack of correlation between personal and familial history of PDAC also highlights the intricacies of germline mutation penetrance and that individuals with such known at-risk susceptibilities may have no affected family members.

Our study has several limitations. Data was collected from a single referral center and only a small number of patients with BRCA1 and BRCA2 genetic mutations were identified despite extensive review of our database extending over an 8-year period. Almost all of the patients included in the study were female. This gender bias was a result of most identified subjects with deleterious BRCA mutations found in the breast cancer genetic counseling clinic registry and breast cancer surgery clinic. Male patients are also carriers of BRCA genes but we were unable to identify any male patient with BRCA mutations in the group with available abdominal imaging. The age and sex characteristics of the included subjects were compared between the groups with and without available abdominal imaging. The results showed comparable age and sex distribution between the two groups which suggest that the small group of 20 patients with available abdominal imaging may represent the bigger group of 66 patients studied with available medical records (Table 3). In addition, our study did not include a matched group of controls without BRCA mutations to compare the frequency of abnormal pancreatic imaging findings.

## 5. Conclusion

Our study shows a high incidence of abnormal pancreatic imaging findings in patients with BRCA1/BRCA2 genetic mutations, including pancreatic atrophy, cysts, ductal dilation, and pancreatic cancer. This incidence was 35% in patients harboring the genetic mutations. Family history of PDAC was more prominent in BRCA2 mutation carriers. Retrospective multicenter studies with large BRCA1 and BRCA2 patient cohorts and prospective studies reviewing serial follow-up imaging on such patients are needed to further assess the potential benefits of modification to current pancreatic cancer screening guidelines.

## Figures and Tables

**Table 1:** Personal and family history of cancer in BRCA1 and BRCA2 mutation carriers.

Patients with available history, *n* = 31	Cancer type	Personal history, *n* (%)	Familial history, *n* (%)
BRCA1	Breast	16 (51.6)	28 (90.3)
	Colon	1 (3.2)	4 (12.9)
	Esophageal	0	1 (3.2)
	Gastric	0	1 (3.2)
	Leukemia	0	1 (3.2)
	Lung	0	4 (12.9)
	Melanoma	0	1 (3.2)
	Ovarian	2 (6.5)	14 (45.2)
	Pancreatic	1 (3.2)	0
	Prostate	0	4 (12.9)
	Renal	0	0

Patients with available history, *n* = 25	Cancer type	Personal history, *n* (%)	Familial history, *n* (%)

BRCA2	Breast	6 (24.0)	22 (88.0)
	Colon	0	6 (24.0)
	Esophageal	0	0
	Gastric	0	2 (8.0)
	Leukemia	0	0
	Lung	0	2 (8.0)
	Melanoma	0	1 (4.0)
	Ovarian	2 (8.0)	12 (48.0)
	Pancreatic	1 (4.0)	4 (16.0)
	Prostate	0	2 (8.0)
	Renal	0	2 (8.0)

**Table 2:** Pancreatic imaging in BRCA1 and BRCA2 mutation carriers.

	Age	Sex	Personal history			Family history			P. atrophy	P. cancer	P. cyst	PD dilation	PD stricture
			Pancreatic Ca	Breast Ca	Ovarian Ca	Pancreatic Ca	Breast Ca	Ovarian Ca					
BRCA1	29	F	−	+	−	−	−	−	−	−	−	−	−
	30	F	−	+	−	−	+	+	−	−	−	−	−
	33	F	−	+	−	−	+	+	−	−	−	−	−
	34	F	−	−	−	−	+	+	+	−	−	−	−
	35	F	−	+	+	−	+	+	+	−	−	−	−
	42	F	−	−	−	−	+	−	−	−	−	−	−
	44	F	−	+	−	−	+	+	−	−	−	−	−
	47	F	−	−	+	−	+	−	−	−	+	−	−
	52	F	−	−	−	−	+	−	−	−	−	−	−
	52	F	−	−	−	−	+	+	−	−	−	−	−
	52	F	−	−	−	−	+	−	+	−	−	−	−
	54	F	−	−	−	−	+	−	−	−	−	−	−
	56	F	−	+	−	−	−	−	−	−	+	−	−
	64	F	+	+	−	−	+	−	−	+	−	+	−

BRCA2	34	F	−	+	−	−	+	+	−	−	−	−	−
	37	F	−	−	−	−	+	−	−	−	−	−	−
	48	F	−	+	−	+	+	−	−	−	−	−	−
	54	F	+	−	−	−	+	−	+	+	−	+	−
	64	F	−	−	+	−	+	−	−	−	−	−	−
	82	F	−	+	+	−	+	−	−	−	−	−	−

**Table 3:** Characteristics of the 66 patients studied based on age and gender.

	Patients with available Imaging		Patients without Imaging	
	(*N* = 20)		(*N* = 46)	
	BRCA1	BRCA2	BRCA1	BRCA2
Age in years	Mean 44.6	Mean 53.2	Mean 43.1	Mean 51.0
	Median 45.5	Median 51.0	Median 38.0	Median 51.0

Gender	Female %	Male %	Female %	Male %
	100.0	0.0	91.3	8.7
